# Measuring self-rated health status among resettled adult refugee populations to inform practice and policy – a scoping review

**DOI:** 10.1186/s12913-017-2771-5

**Published:** 2017-12-08

**Authors:** Alison Dowling, Joanne Enticott, Grant Russell

**Affiliations:** 1 School of Primary Health Care, Monash University, Melbourne, VIC Australia; 2 Department of Psychiatry, Southern Synergy, Monash University, Melbourne, VIC Australia

**Keywords:** Refugees, Asylum seekers, Settlement, Humanitarian, Self-perceived health, Subjective health, Surveys and questionnaires, Measures, Tools and instruments

## Abstract

**Background:**

The health status of refugees is a significant factor in determining their success in resettlement and relies heavily on self-rated measures of refugee health. The selection of robust and appropriate self-rated health measurement tools is challenging due to the number and methodological variation in the use of assessment tools across refugee health studies. This study describes the existing self-report health measures which have been used in studies of adult refugees living in the community to allow us to address the challenges of selecting appropriate assessments to measure health within refugee groups.

**Methods:**

Electronic databases of Ovid Medline, CINAHL, SCOPUS, Embase and Scopus.

**Results:**

This review identified 45 different self-rated health measurements in 183 studies. Most of the studies were cross sectional explorations of the mental health status of refugees living in community settings within Western nations. A third of the tools were designed specifically for use within refugee populations. More than half of the identified measurement tools have been evaluated for reliability and/or validity within refugee populations. Much variation was found in the selection, development and testing of measurement tools across the reviewed studies.

**Conclusion:**

This review shows that there are currently a number of reliable and valid tools available for use in refugee health research; however, further work is required to achieve consistency in the quality and in the use of these tools. Methodological guidelines are required to assist researchers and clinicians in the development and testing of self-rated health measurement tools for use in refugee research.

## Background

Globally, the number of people forcibly displaced by conflict is at the highest levels ever recorded. At the end of 2015, it was reported that the number of refugees had reached 21.3 million [1]; an increase of 1.7 million from 2014 [2]. A further 3.2 million were asylum seekers [1]. Conflicts, violence and human rights violations, particularly in the Middle East and North Africa, are forcing millions of people to leave their homes and to flee from destruction and persecution. A refugee has a well-founded fear of persecution for reasons of race, religion, nationality, political opinion or membership in a particular social group [1].

Resettlement to a third country may be offered to those refugees who cannot return to their home country for fear of persecution or when they cannot be offered a permanent residence in the country they are currently living [3]. Resettling refugees are taking on added importance internationally with massive movements of people across continents due to global political and economic instability. The long term health and settlement prospects of refugees are a matter of continuing relevance for receiving nations as they are recognized as one of the most vulnerable groups in our society in terms of risk for poor health [4–10]. They often have unique health needs reflecting the epidemiology of diseases in their country of origin [11]; inadequate and disrupted health care; and stressors experienced during the migration and resettlement periods; including trauma, torture and poverty. Exposure to pre-migration trauma may have a lasting impact on their psychological and physical well-being and high levels of stress due to assimilation into a new society may also contribute to the health of resettled refugees [12–18].

The collection of refugee health data relies heavily on subjective measures of refugee health because they are less resource intensive than clinical assessments, less burdensome on participants and can capture individual perceptions of health, such as psychosocial factors geographic location and individual characteristics [19]. A large number of empirical studies have demonstrated that a person’s own appraisal of his/her general health is a powerful predictor of future morbidity and mortality, even after controlling for a variety of physical, socio-demographic and psycho-social health status indices [20–25]. Therefore, measurement of self-rated health within resettling refugees may well serve as a surrogate for more traditional clinical assessments of health. Despite this, the selection of robust and appropriate subjective measurement tools for use among this population group is challenging due to methodological variation in the use of assessment tools across refugee health studies. This has meant that refugee health data is often conflicting and difficult to interpret and compare. Several studies have reviewed health measurement tools used in refugee populations [26–29] and all have suggested that the measurement tools being used in refugee health research often lack the validity and rigour required to assess constructs of psychological wellbeing, health and other factors that are associated with resettlement outcomes.

This review will build on these earlier reviews by providing an update on the health measurement tools being used in adult refugee research. However, in contrast with these earlier systematic studies, this review will not be limited by specific health concepts; such as trauma [26, 28]; refugee gender [27], and we will include refugees living within western and non-western nations; allowing a broader investigation of the literature reporting the use of these measurement tools. The development of such a comprehensive knowledge base is required in the literature; particularly at this time when refugee movement across the globe is unpresented and receiving nations are faced with increased pressure to provide immediate and long term health care that is appropriate, effective and comparative.

## Study aim

The aim of this scoping review is to describe the self-report health measures which have been used in studies of adult refugees living in the community. For each self-report measure identified, we will note any reliability and validity testing within refugee groups as well as the settings in which the measures have been used. By doing this, we aim to gain a better understanding of how these measures are used in refugee health research. This will allow us to address the challenges of selecting appropriate assessments to measure health within refugee groups.

## Methods

A scoping review was conducted between October 2016 and February 2017. Scoping reviews are rigorous, with methods that allow for replication, but findings are not synthesized or aggregated to the extent customary in systematic reviews. They allow for the inclusion of diverse study designs and involve iterative search process where search terms may evolve during the review [30]. A scoping review can help to identify gaps in the evidence base and summarize a more broad range of research findings [30]. We adopted the Arksey and O’Malley (2005) five stage methodological framework for scoping reviews:

Stage 1:    Identifying the research question
Stage 2:    Identifying the relevant studies
Stage 3:    Selecting studies
Stage 4:    Charting the data
Stage 5:    Collating, summarizing and reporting the results

## Stage 1: Identifying the research question

Our research question was: *“*What is known about the measurement tools used to measure self-rated health within resettled refugee groups?”

More specifically, the present review aims to address the following questions:

1. What settings have been described in studies that have measured self-rated health among refugee populations?
2. What self-rated health measurement tools have been used in these studies?
3. Which of these self-rated health measurement tools been evaluated for validity and reliability criteria within refugee populations?

## Stage 2: Identifying the relevant studies

We searched five electronic databases (Medline, CINAHL, EMBASE, SCOPUS and PsychINFO) for English language papers published between January 2000 to March 2017. The following search terms were employed: “refugee”, “asylum seeker”, “settlement”, “humanitarian”, “self-perceived health”, “subjective health”, “mental health”, “mental disorder”, “physical health”, “health status”, “surveys and questionnaires”, “scales”, “screening”, “measures” and “instruments”. The initial searches were performed in December 2016 and subsequently re-run in Medline in March 2017 to identify additional relevant studies published in 2016 and 2017. No additional studies were identified. This search was supplemented with a general Internet search using Google and Google Scholar to ensure our results were maximal.

### Inclusion and exclusion criteria

For a measurement tool to be included in the review, two eligibility steps were required. First, published peer-reviewed articles were required to meet the following criteria:

1. Published in the English language.
2. Published between January, 2000 and March, 2017.
3. Focused on the collection of self-rated physical and mental health from refugees or asylum seekers using specific health assessment tools.
4. Focused on adults (defined as those aged 15 years and above)
5. Focused on community living refugees and asylum seekers; including those living within refugee camps which offer long-term or permanent settlement, and located outside of the refugee or asylum seeker’s home country.
6. Included different population groups other than those of interest (e.g. immigrants) but reported refugee and asylum seeker data separately.
7. Studies were excluded if they were classified as an incomplete article (e.g. Editorial, commentary, letter or conference abstract); were review articles or reported data already used in another included article.

Second, the measurement tool(s) reported in the articles using eligibility criteria 1–7 were required to meet the following inclusion criteria:

8. Had been specifically tested for validity and/or reliability within refugee groups. (Given the limited volume of research in this area, the search was not limited by date of publication).
9. Had not been specifically tested for validity and/or reliability within refugee groups but were described in studies where:

- the refugee sample size was ≥ 150; or
- the tool(s) were described in 5 or more research articles.

These tools were included based on their use in research studies of what the study authors considered large sample sizes or in an acceptable number of research studies.

## Stage 3: Selecting studies

**Fig. 1:**
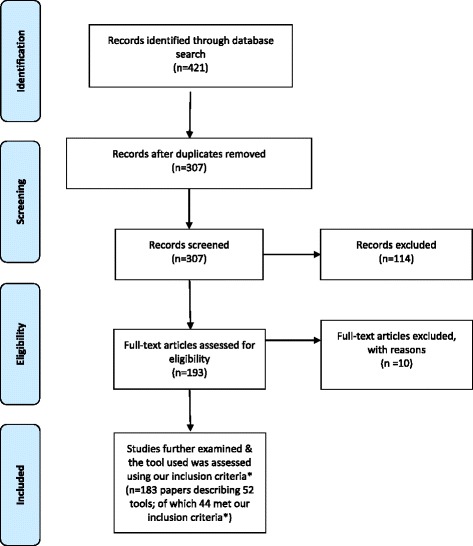
PRISMA diagram: search and selection process. *Tools/instrument(s) were assessed as meeting the inclusion criteria if they reported being tested for validity and/or reliability within refugee groups, or if the refugee sample size was >150, or if described in 5 or more research articles

The screening and selection procedure is shown in Fig. 1 using the Preferred Reported Items in Systematic Reviews and Meta-analysis (PRISMA) flowchart [31]. It was difficult to ascertain between projects and papers (i.e. projects are those where multiple papers were published from the one study as opposed to single studies from single projects) and based on this, the decision was made to look at papers, not projects, for the review.

A total of 390 references were obtained from the initial search of which 114 studies were duplicates. One author (AD) screened the remaining 276 references, applying the inclusion criteria to the titles and abstracts where possible. Full text review was conducted on 193 articles with a further 10 articles excluded because they did not report on resettled refugees; were studies that included different population groups other than those of interest (e.g. immigrants) but did not report refugee and asylum seeker data separately; did not specify the name of the measurement tool(s) used to collect refugee health data; or were studies researching the health of internally displaced refugees. The final set of articles was 183 papers with 52 measurement tools identified. To reduce bias caused by human error, two authors (GR and JE) repeated 10% of the study selection process. Rates of agreement were consistently high between the three reviewers, with any discrepancies resolved through discussion.

## Stage 4. Charting the data

The 183 articles were charted in Microsoft Excel 2010, using the study characteristics (e.g. author information, publication year, study design), participant characteristics (e.g. demographic data), measurement tool name and statistical outcome information (e.g. measurement tool validation and reliability data).

## Stage 5: Collating, summarizing and reporting the results

We devised the following categories of focus: self-rated health measurement tool name and description; health variable (eg. mental health, general health); study setting (eg. clinic, community, refugee camp); study population (eg. refugee, asylum seeker); and reliability/validation data regarding the measurement tool(s) conducted within refugee or asylum seeker populations. Common themes were identified across articles, and when possible, articles were compared.

## Definitons

For the purpose of this scoping review, the following definitions have been applied.

### Study population

Refugees and asylum seekers included in this study are those who were resettled in a community in a country outside of their own. This included those who had been offered permanent residency in a third country (either through United Nations High Commission for Refugees (UNHCR) mandate; permanent visas) or were living in a refugee camp for over 12 months.

### Self-rated health measurement

Self-rated health measures are defined as any report on the status of an individual’s health condition that comes directly from the individual, without interpretation of the individual’s response by a clinician, researcher or anyone else [32]. Modes of data collection include interviewer-administered measurement tools, self-administered measurement tools, computer-administered measurement tools or interactively administered measures [33].

### Reliability

Reliability was defined as to the extent to which scores are the same for repeated measurement under different conditions [34].

### Validity

Validity is the extent to which a tool measures what it is supposed to measure and performs as it is designed to perform [34].

## Results

### The studies

A total of 183 articles were reviewed for this study (see Table 1 for a summary). Most of the studies (123 in total) were conducted within resettled refugees living in community settings within Western nations. Studies investigating the health of refugee populations living in non-metropolitan areas were limited, apart from 19 studies which were conducted in refugee camps. A large proportion of the research focused on populations from the Middle East, although Syrian refugees were under-represented with only 5 studies investigating the health status of this refugee group. Refugee mental health status was the focus of most of the reviewed studies (*n* = 153); particularly symptoms of depression, anxiety and post-traumatic stress disorder (PTSD). Studies were predominantly cross sectional in design and comprised of sample sizes of less than 500 participants. Recruitment mainly involved both sexes with the exception of 10 studies where only females were invited to participate and 2 studies where only males were recruited. The measurement tools were administered to participants using several methods; including face to face oral administration by trained interviewers/interpreters and self-completion.

**Table 1:** Self-rated health study characteristics

Characteristic	# studies
	(*N* = 183)
Country:	
Western	
United States	59
Australia	20
Sweden	14
Norway	8
Canada	7
UK, Denmark, The Netherlands	6
New Zealand	4
Finland, Sth Korea	3
Ireland, Switzerland, Germany	2
Other	6
Non Western	
India, Uganda	4
Nepal, Rwanda	3
The Gambia, Jordan	2
Other	15
Setting:	
Non-clinical	135
Clinical	48
Health assessment	
Mental Health	153
General Health	30
Gender:	
Mixed sex sample	171
Female only sample	10
Male only sample	2
Sample size:	
< 500	163
500–1000	12
> 1000	8
Population/region	
Middle East	72
Sth East Asia	58
Sub-Saharan Africa	52
Eastern Europe	35
Study Design:	
Cross sectional	173
Longitudinal	9
Case control	1
Data type:	
Primary data collection	179
Secondary data analysis	4

### The measurement tools

A total of 52 tools were identified in the retrieved studies, of which 45 met the study inclusion criteria (see Tables 2 and 3 for characteristics of these measurement tools). Seven tools were excluded because they had not been specifically tested for validity and/or reliability within refugee groups; were reported in studies where the refugee sample size was ≤150 or where the measurement tool(s) were described in less than 5 research articles.

**Table 2:** Overview of the self-rated health measurement tools identified in the scoping review

Measurement focus	Measurement name, Author & Year	Design/use	No. of studies tool cited in	Composition	Completion time (CT); Language Availability (LA)	Publically available	Reliability testing in refugee population group	Validity testing in refugee population group
General Health	Medical Outcomes Study Short Form - Ware & Sherbourne (1992)	Designed for use in clinical practice and research, and general population surveys.	18	Several versions: 36, 20, 12 or 6 items measuring perceived mental & physical health status across 8 health concepts in past month.	5–10 min to complete depending on version. Available in 50 languages.	Yes	SF-6: Afghani^(1)^ SF-12: Russian^(2)^ SF-20: Bosnian^(3)^ SF-36: Bosnian^(4)^	SF-12: Russian^(2)^
General Health	WHO Quality of Life-BREF - WHO (1998)	Designed to assess self-rated quality of life across different cultures. Appropriate for large research studies, clinical trials, medical care.	6	26 items measuring 4 quality of life domains (physical health, psychological health, social relationships & environment) in past 2 weeks. Responses scored on 5-point Likert scale.	10–15 min to complete. Available in multiple languages	No	Somalian^(5)^. Liberian, Togolese, Sierra-Leonean^(6)^.	Somalian^(8)^
General Health	New Mexico Refugee Symptom Checklist-121 - Hollifield et al. (2009)	Designed for use within non-clinical refugee research.	2	121 items; 12 subscales assessing distressing physical and emotional symptoms in past year. Responses scored on 5-point Likert severity scale.	60–90 min to complete. Available in multiple languages	No	Kurdish & Vietnamese^(7)^	Kurdish & Vietnamese^(7)^
General Health	Personal Wellbeing Index - Cummins (2003)	Designed to be a cross-cultural instrument of quality of life.	2	8 items assessing life satisfaction (eg.health, community belonging, future security, religion & spirituality). Responses scored on 11 point Likert satisfaction scale.	5–10 min to complete. Available in multiple languages	Yes	Afghani & Kurdish^(8)^	No
General Health	World Health Organisation Disability Assessment Schedule 2.0 - WHO (2004)	Designed to be used within clinical, community and general populations.	1	36 items measuring quality of life (eg. mobility, self-care, interpersonal). Responses scored on 5-point Likert scale.	5–20 min to complete. Available in multiple languages.	No	Sudanese^(9)^	No
Common Mental Disorders	Hopkins Symptom Checklist-25 - Mollica et al. (1987)	Originally designed for use in Western clinical settings. Indochinese version developed to screen anxiety & depression in clinical refugee samples.	62	25 items; 15 anxiety and 10 depression symptoms. Responses scored on a 4-point scale. An average-item score > 1.75 indicates clinically significant distress.	5–10 min to complete. Available in multiple languages (eg. English, Amharic, Bosnian, Cambodian, Dari, Khmer, Laotian, Pashto, Vietnamese, Tibetan)	No	African (10) (11, 12) (13) (14) (15) (16) Nth & Sth East Asian (17)(18)(19)(20)(21)(22)(25) Middle Eastern (12)(13)(14)(18)(19)(20) Southern & Eastern Europe (24)(12)(13)(14)(22)(18)(3) Central American (13)(19)	Tibetan^(17)^. Cambodian, Laotian, Vietnamese^(18)^. Syrian^(20)^.
Common Mental Disorders	General Health Questionnaire - Goldberg & Blackwell (1970)	Designed for identifying psychiatric illness in Western general practice patients.	11	Several versions: 60, 30, 28 or 12 items measure depression, anxiety, social impairment, & hypochondriasis. Responses scored on 4 point Likert scale indicating presence or absence of symptoms. Cutoff scores vary according to version.	2–10 min to complete depending on version. Available in 38 languages.	No	Somalian^(21)^ Bosnian^(22)^ Kosovan^(23)^ Bhutanese^(24)^	No
Common Mental Disorders	Centre for Epidemiologic Studies-Depression Scale - Radloff et al. (1977)	Designed to measure current depressive symptoms in general populations.	10	20-item measure assessing symptoms of depression in past week. Responses scored on 4-point Likert scale. Suggested cut-off >16.	10–20 min to complete. Available in multiple languages.	Yes	Iraqi^(25)^ Syrian^(28)^ Iraqi, Lebanese, Yemenese^(26)^ Arabic^(27)^ Bosnian^(28)^	No
Common Mental Disorders	Beck Depression Inventory - Beck (1961)	Designed to detect/assess/monitor changes in depressive symptoms in clinical setting by trained professionals.	9	21 items of depression rated from 0 to 3 in terms of intensity. Suggested cut offs: minimal depression <10; mild-moderate depression 10–18; mod to severe depression 19–29; severe depression 30–63.	10–15 min to complete. Available in multiple languages.	Yes	Hmong^(29)^ Syrian^(30)^ Somalian^(29)^	Hmong^(37)^
Common Mental Disorders	Kessler Psychological Scale - Kessler & Mroczek (1994)	Developed to screen for distress/mental illness in Western populations. Has been adopted in Western and non-Western countries as a screening & outcome measure in primary care and mental health settings.	8	10 or 6 items measuring psychological distress over previous 4 weeks. Responses scored on 5 point Likert scale. Recommended cut-off scores: low to mild distress (10–20); moderate (22–29); severe (> = 30).	10 mins to complete. Available in multiple languages.	Yes	Iraqi^(31, 32)^ Kurdish^(39)^ Afghani^(33)^ Malian^(34)^	No
Common Mental Disorders	Symptom Checklist-90 - Derogatis & Cleary (1977)	Designed as mental health screening tool for Western clinical samples.	8	90 items; 10 subscales asking about anxiety, depression, somatization, hostility, interpersonal sensitivity, phobic anxiety, paranoid ideation, obsessive-compulsive ideation, psychoticism in past week. Responses scored on 5 point scale of distress.	15–30 min to complete. Available in multiple languages.	No	No	Hmong^(35)^
Common Mental Disorders	Self-Reporting Questionnaire - WHO (1995)	Designed as psychiatric screening instrument for use in primary health care and/or community settings in low/middle income countries.	5	Depending on version, Items range from 5 to 20 asking about psychological and somatic symptoms in the past 30 days. Optimal cut-off considered to be 7/8.	5–10 min to complete. Available in multiple languages.	Yes	No	No
Common Mental Disorders	Internalized Stigma of Mental Illness Scale - Ristcher et al. (2003)	Designed to measure internalized stigma within Western clinical samples.	3	29 items; 5 subscales measuring subjective experience of stigma. Responses scored on 4 point Likert scale. Higher total scores indicate greater internalized stigma.	Completion time: n/a. Available in multiple languages.	Yes	Iraqi, Lebanese, Yemeni^(39)^	Iraqi, Lebanese, Yemeni^(39)^
Common Mental Disorders	Afghan Symptom Checklist - Miller et al. (2006)	Designed to measure psychological distress within non-clinical samples of Afghanis.	2	23 items measuring well-being and distress in the previous 2 weeks. Responses scored on 5 point Likert scale.	10 mins to complete. Available in English & Dari.	No	Afghani^(1)^	No
Common Mental Disorders	Hospital Anxiety & Depression Scale - Zigmond & Snaith (1983)	Designed for use in Western clinical & research settings.	2	14 items measuring cognitive & emotional aspects of depression and anxiety. Responses on 4-point Likert scale.	5 mins to complete. Available in multiple languages.	No	No	No
Common Mental Disorders	Augmented Anxiety Sensitivity Index -Hinton et al. (2006)	Designed to measure culturally specific fears within Cambodian refugee outpatients in USA.	1	ASI: 16 items assessing fear of different anxiety symptoms. Responses scored on 5-point Likert scale. Augmented ASI: ASI with 9-item addendum assessing anxiety-related concerns.	Completion time: n/a. Available in English & Cambodian.	No	Cambodian^(36)^	No
Common Mental Disorders	Beck Anxiety Inventory - Beck (1988)	Designed to measure anxiety in adults and adolescents in clinical & research settings.	1	21 items of anxiety rated from 0 to 3 with total possible score of 63. Suggested cut-offs: 0–7: minimal anxiety; 8–15: mild anxiety; 16–25: moderate anxiety; >26: severe anxiety.	5–10 min to complete. Available in multiple languages.	Yes	No	No
Common Mental Disorders	Depression, Anxiety Stress Scale - Lovibond & Lovibond (1995)	Designed to measure symptoms of depression, anxiety & stress in community settings.	1	20-item measure assessing symptoms of depression in past week. Responses scored on a 4-point Likert scale. Suggested cut-off >16.	10–20 min to complete. Available in multiple languages.	Yes	Arabic^(34)^	No
Common Mental Disorders	General Perceived Self-Efficacy Scale - Schwarzer & Jerusalem (1995)	Designed to assess self-efficacy within Western non-clinical/clinical populations.	1	10 items assessing ability to cope with daily hassles and flexibility to adapt after experiencing stressful life event(s). Responses scored on 4 point Likert scale. Higher scores indicate stronger patient’s belief in self-efficacy.	2–10 min to complete depending on version. Available in multiple languages.	No	Afghani, Kurdish^(8)^	No
Common Mental Disorders	Mental Health Inventory - Veit & Ware (1983)	Designed for use in general Western populations to detect mental health disorders.	1	18 items, consisting of two subscales: psychological distress (22 items) and psychological wellbeing (16 items). Responses scored on 6-point Likert scale. Higher scores reflect better mental health.	5–10 min to complete. Available in multiple languages.	Yes	Bosnian^(37)^	No
Common Mental Disorders	Patient Health Questionnaire - Spitzer & Williams (1994)	Designed to measure depression & severity in Western clinical samples.	1	9 items measuring depressive disorder according to the DSM-IV. Responses scored on 4-point Likert scale indicating degree of severity.	5 mins to complete. Available in multiple languages.	No	No	No
Common Mental Disorders	Refugee Health Screener-15 -- Hollifield et al. (2013)	Designed to screen common mental disorders in clinical samples of refugees.	1	15 items assessing distress, anxiety and depression. Items include 1 coping item, 13 symptom items and 1 distress thermometer. Responses scored on 5 point scale. Suggested cut-offs >12 or >5 on distress thermometer (postive cases).	5–10 min to complete. Available in 11 languages.	Yes	Burmese, Bhutanese, Iraqi^(38)^	Burmese, Bhutanese, Iraqi^(46)(39)^
Common Mental Disorders	Sheehan Disability Inventory - Sheehan (1983)	Designed to assess functional impairment associated with anxiety disorder diagnosis in Western clinical samples.	1	3 items measuring impairment due to the disruption of daily activities in the areas of work, social life, family and home life due to current psychiatric symptoms. Responses scored on analogue scale from 1 to 10 with 10 being the lowest possible level of functioning.	5–10 min to complete. Available in 48 languages.	Yes	No	No
Common Mental Disorders	Vietnamese Depression Scale -Kinzie et al. (1982)	Designed to measure depression among clinical samples of Vietnamese refugees.	1	15 items assessing depression and physical & psychological symptoms associated with depression. 6 depression items are culturally specific for Vietnamese population. Uses 3- and 4-point Likert scales. Optimal cut-off ≥13.	5–10 min to complete. Available in English & Vietnamese.	No	Vietnamese^(40, 41)^	Vietnamese^(48)(49)^
Trauma/PTSD	Harvard Trauma Questionnaire -Mollica et al. (1992)	Designed for clinical samples of refugees. Must be modified and adapted to the characteristics of each cultural group.	62	4 parts: Part 1-measures 17 trauma items. Part 2- respondent describe most traumatic experience/event experienced during their refugee experience. Part 3- assesses likelihood of head injury. Part 4-measures 30 trauma symptoms. Scores of 2.5 or above considered “positive” for PTSD in Indochinese populations.	50–60 min to complete. Available in multiple languages.	No	African (11)(12)(14)(15)(16)(42) (43) Nthn & Sth East Asian (11)(17)(20)(22)(44)(45)(46) Middle Eastern (12)(14)(21)(24)(44)(45)(54) Sthn & Eastern Europe^(12)(44)(14)(45)(17)^ Central American^(27)^	Cambodian, Laotian & Vietnamese^(52)(53)^ Sub-Saharan Africa^(50)^ Chechen & Afghan^(12)^
Trauma/PTSD	Posttraumatic Diagnostic Scale (PDS) -- Foa et al. (1996)	Designed to provide brief self-report measure of PTSD in both clinical and research settings.	15	4 parts. Part 1-checklist of traumatic events that respondent may have experienced or witnessed. Part 2-Respondents indicate which event has disturbed them the most in past month. Part 3–17 PTSD symptoms items experienced in past month. Responses scored on 4-point Likert scale (0-not at all or only one time to 3-five or more times a week or almost always).	20 mins to complete. Language availability: English	Yes	Turkish, Iranian, Sri-Lankan, Bosnian, Afghani^(46)^ Iraqi^(23)(46)^ Asian, European, African^(46)^	Iraq, Lebanese, & Arab^(33)^ Ugandan & Somalian^(47)^ Syrian^(28)^ Asian, European, & African^(60)^ Kosovar^(48)^ Rwandan^(49)^
Trauma/PTSD	Post Migration Living Difficulties Scale - Silove et al. (1998)	Designed to assess current life stressors of asylum-seekers.	10	23 items to assess recent adverse life experiences typical of migration (eg. delays in processing applications, access to health & welfare, & stressor elated to resettlement). Responses scored on 5-point scale.	10–20 min to complete. Languages: English.	No	No	Tamil^(50)^
Trauma/PTSD	Posttraumatic Symptom Disorder Checklist - Weathers et al. (1993) PCL-90	Designed to measure PTSD among non-clinical sample in USA.	7	17 items assessing DSM-IV PTSD symptoms. Responses on 5 point Likert scale as to how much these have been bothered by each item in past month. Some training is required to administer test.	5–10 min to complete. Language availability: English,	Yes	No	Somali & Oromo^(51)^ Darfuri^(52)^
Trauma/PTSD	Impact of Events Scale-Revised - Horowitz et al. (1979)	Designed to measure current subjective distress related to a specific event among Western clinic samples.	5	15 items related to a particular event. 7 items measure intrusiveness & 8 items measure avoidance. Responses scored on 4-point Likert scale according to how often each item has occurred in past 7 days.	45–60 min to complete. Available in multiple languages.	Yes	Chechyan, Afghani & West African^(12)^. Middle Eastern^(53)^. Bosnian^(54)^. Middle East, Central Africa, Sth Asia & Sth E Europe^(10)^. Somalian^(55)^	Chechyan, Afghani & West African^(12)^
Trauma/PTSD	Comprehensive Trauma Inventory-104 - Hollifield et al. (2005)	Designed for use within community samples of refugee.	4	104 items; 12 scales assessing range of traumatic events (eg psychological & physical injury, detention, intentional abuse, deprivation, discrimination). Responses scored on 5-point Likert scale whether or not the event was experienced and, if so, how much impact the event had.	60 mins to complete. Available in English, Kurdish, Vietnamese.	Yes	Kurdish & Cambodian^(56)^	Kurdish & Cambodian^(70)^
Trauma/PTSD	Cumulative Trauma Disorder Scale - Kira et al. (2012)	Designed for use within clinical & non-clinical samples of refugees.	4	15 items measuring trauma symptoms (eg depression, anxiety, somatization) in previous month. Responses on 5 point scale.	10 mins to complete. Languages: English,	No	Iraqi, Lebanese & Yemeni^(57)^ Arabic^(34)^	Iraqi^(71)^
Trauma/PTSD	HADStress - Westermeyer et al. (2010)	Designed to measure somatic symptoms within community sample of Somali refugees living in USA.	4	4 items measuring somatic symptoms: headaches, appetite change, dizziness and sleep problems. Responses on 0–4 scale with higher scores indicating more somatic symptoms. Cut off score of >3 recommended.	10 mins to complete. Languages: English,	No	No	Somalian^(58)^ Ethiopian^(59)^
Trauma/PTSD	Post Traumatic Growth Inventory - Tedeschi & Calhoun (1996)	Designed to assess positive outcomes among traumatized non- clinic Western samples.	3	21 item assessing positive outcomes among those who have experienced traumatic events. Responses scored on 6 point scale.	5–10 min to complete. Available in multiple languages.	Yes	Arabic^(67)^ Yugoslavian(60)	Arabic^(67)^ Bosnian^(61)^
Trauma/PTSD	Posttraumatic Symptom Scale - Holen et al. (1983)	Designed in sample of German Armed Forces following a UN peace keeping mission to Cambodia to determine causes and scale of stress reactions.	3	10 or 16 items measuring PTSD symptoms in past 7 days according to DSM-III criteria. Presence or absence of symptoms is rated in Yes/No format. Sum of positive items used as total score.	10 mins to complete. Languages: English	Yes	Bosnian^(62)^	No
Trauma/PTSD	Annihilation Anxiety Scale - Kira et al. (2012)	Designed for use in clinical settings as well as in research with severely traumatized populations, such as refugees.	2	3 items that assess annihilation anxiety. Responses are scored on 5 point Likert scale.	5 mins to complete. Languages: English,	No	Iraqi, Lebanese & Yemeni^(34)^	Iraqi, Lebanese & Yemeni^(34)^
Trauma/PTSD	Health Leaflet -Sondergaard et al. (2003)	Designed to measure PTSD within community samples of refugees	2	15 items to screen for PTSD. Yes/No responses to items consisting of psychological symptoms, network and attitude towards the future in the new country and persecution. Suggested cut off = 10.	10 mins to complete. Languages: n/a.	No	No	Iraqi^(63)^
Trauma/PTSD	Minnesota Multiphasic Personality Inventory-2 and Minnesota Multiphasic Personality Inventory-2-RF - Tellegen & Ben-Porath (2008)	Designed to assess personality traits and psychopathology among Western clinic samples.	2	MMPI-2: 567 items measuring personality and psychopathology, consisting of 8 validity scales, 10 clinical scales & 15 content scales. Responses are recorded as True/False. MMPI-2-RF: 338 items from MMPI-2, including 9 validity scales.	25–90 min to complete depending on version. Available in multiple languages.	Yes	No	Korean^(64)^ Vietnamese^(65)^
Trauma/PTSD	Traumatic Life Events Questionnaire - Kubany (2000)	Designed in general clinic western population for use in research, primary care or emergency rooms.	2	23-items measuring 22 types of potentially traumatic events. Responses scored on Likert scale range from “never” to “more than 5 times” and whether fear, helplessness or horror was present (“yes/no”).	10–15 min to complete. Available in English & Spanish.	No	No	No
Trauma/PTSD	Cambodian Somatic Symptom & Syndrome Inventory - Hinton et al. (2013)	Designed for use in clinical samples of Cambodian refugees. Very specific to Cambodians.	1	23 items; 2 subscales assessing somatic symptoms & cultural syndromes in past week. Somatic subscale (12 items) & syndrome scale (11 items). Responses scored on 5 point Likert scale asking how much he/she was bothered by certain somatic symptoms & syndromes.	10 mins to complete. Languages: English & Cambodian.	No	Cambodian^(66)^	Cambodian^(80)^
Trauma/PTSD	Dissociative Experiences Scale - Carlson & Putnam (1986)	Designed to measure dissociation in normal & clinic Western populations	1	28 items that assesses dissociative symptoms. Each item asks about the frequency (from Never to Always) that a particular dissociative symptom is experienced. Scores of >20 indicate probable posttraumatic or dissociative disorders.	10 mins to complete. Available in multiple languages.	Yes	Ethiopian^(51)^ Bosnian^(67)^	No
Trauma/PTSD	Posttraumatic Stress Disorder Interview for Vietnamese Refugees - Doa et al. (2012)	Designed to assess trauma among clinic samples of Vietnamese refugees.	1	Part A: 30 items assessing pre-migration, migration, encampment, post-migration traumas. Yes/No response. ‘Yes’ response to Part A item(s), respondent proceeds to Part B which assesses PTSD criteria.	Completion time: n/a. Available in English & Vietnamese.	No	Vietnamese^(68)^	Vietnamese^(82)^
Trauma/PTSD	Primary Care Post-Traumatic Stress Disorder Screen - Prins et al. (2003)	Designed for use in primary care and other medical settings.	1	5 items assessing dimensions of PTSD (re-experiencing, numbing, avoidance, hyperarousal) with Yes/No response options.	5 mins to complete. Language availability: English,	Yes	No	No
Trauma/PTSD	Resettlement Stressor Scale - Clarke et al. (1993)	Designed to measure stressors faced within a community sample of Cambodian refugee adolescents.	1	32 items assessing trauma events in migration/present settlement. Responses scored on 5-point scale.	10–20 min to complete. Avail in English & Cambodian.	Yes	No	Cambodian^(69)^
Trauma/PTSD	War Trauma Scale -Clarke et al. (1993)	Designed for use within clinical samples of Cambodian adolescent refugees that had lived through the Pol Pot regime.	1	42 items measuring traumatic experiences inflicted by Pol Pot regime. Items are all worded in relation to Pol Pot era.	20–30 min to complete. Available in English & Cambodian.	No	Cambodian^(83)^	Cambodian^(83)^

References

^1^Alemi Q, Stempel C, Baek K, Lares L, Villa P, Danis D, et al. Impact of Postmigration Living Difficulties on the Mental Health of Afghan Migrants Residing in Istanbul. International Journal of Population Research. 2016;2016

^2^Hoffmann C, McFarland BH, Kinzie JD, Bresler L, Rakhlin D, Wolf S, et al. Psychometric properties of a Russian version of the SF-12 Health Survey in a refugee population. Comprehensive psychiatry. 2005;46(5):390–7

^3^Mollica RF, Caridad KR, Massagli MP. Longitudinal study of posttraumatic stress disorder, depression, and changes in traumatic memories over time in Bosnian refugees. The Journal of nervous and mental disease. 2007;195(7):572–9

^4^Weine SM, Razzano L, Brkic M, Ramic A, Miller K, Smajkic A, et al. Profiling the Trauma Related Symptoms of Bosnian Refugees Who Have not Sought Mental Health Services. The Journal of Nervous and Mental Disease. 2000;188(7):416–21

^5^Redko C, Rogers N, Bule L, Siad H, Choh A. Development and validation of the Somali WHOQOL-BREF among refugees living in the USA. Quality of life research: an international journal of quality of life aspects of treatment, care and rehabilitation. 2015;24(6):1503–13

^6^Akinyemi O, Owoaje E, Ige O, Popoola O. Comparative study of mental health and quality of life in long term refugees and host populations in Oru-Ijebu, Southwest Nigeria. BMC Research Notes. 2012;5:394

^7^Hollifield M, Warner TD, Krakow B, Jenkins J, Westermeyer J. The range of symptoms in refugees of war: the New Mexico Refugee Symptom Checklist-121. The Journal of nervous and mental disease. 2009;197(2):117–25

^8^Sulaiman-Hill CMR, Thompson SC. Selecting instruments for assessing psychological wellbeing in Afghan and Kurdish refugee groups. BMC Research Notes. 2010;3

^9^Rasmussen A, Nguyen L, Wilkinson J, Vundla S, Raghavan S, Miller KE, et al. Rates and Impact of Trauma and Current Stressors Among Darfuri Refugees in Eastern Chad. American Journal of Orthopsychiatry. 2010;80(2):227–36

^10^Schubert CC, Punamki R-L. Mental health among torture survivors: cultural background, refugee status and gender. Nordic Journal of Psychiatry, 2011, Vol65(3), p175–182. 2011;65(3):175–82

^11^Keller A, Lhewa D, Rosenfeld B, Sachs E, Aladjem A, Cohen I, et al. Traumatic experiences and psychological distress in an urban refugee population seeking treatment services. Journal of Nervous & Mental Disease. 2006;194(3):188–94

^12^Renner W, Salem I, Ottomeyer K. Cross-cultural validation of measures of traumatic symptoms in groups of asylum seekers from Chechnya, Afghanistan, and West Africa. Social Behavior and Personality. 2006;34(9):1101–14

^13^Teodorescu D-S, Siqveland J, Heir T, Hauff E, Wentzel-Larsen T, Lien L. Posttraumatic growth, depressive symptoms, posttraumatic stress symptoms, post-migration stressors and quality of life in multi-traumatized psychiatric outpatients with a refugee background in Norway. Health and quality of life outcomes. 2012;10:84

^14^Jakobsen M, Thoresen S, Johansen LEE. The Validity of Screening for Post-traumatic Stress Disorder and Other Mental Health Problems among Asylum Seekers from Different Countries. Journal of Refugee Studies. 2011;24(1):171–86

^15^Bentley JA, Thoburn JW, Stewart DG, Boynton LD. The indirect effect of somatic complaints on report of posttraumatic psychological symptomatology among Somali refugees. Journal of Traumatic Stress. 2011;24(4):479–82

^16^Schweitzer R, Melville F, Steel Z, Lacherez P. Trauma, post-migration living difficulties, and social support as predictors of psychological adjustment in resettled Sudanese refugees. Australian & New Zealand Journal of Psychiatry. 2006;40(2):179–87

^17^Lhewa D, Banu S, Rosenfeld B, Keller A. Validation of a Tibetan translation of the Hopkins Symptom Checklist 25 and the Harvard Trauma Questionnaire. Assessment. 2007;14(3):223–30

^18^Corvo K, Peterson J. Post-traumatic stress symptoms, language acquisition, and self-sufficiency: a study of Bosnian refugees. Journal of Social Work. 2005;5(2):205–19

^19^Sabin M, Lopes Cardozo B, Nackerud L, Kaiser R, Varese L. Factors associated with poor mental health among Guatemalan refugees living in Mexico 20 years after civil conflict. Jama. 2003;290(5):635

^20^Weinstein N, Khabbaz F, Legate N. Enhancing need satisfaction to reduce psychological distress in Syrian refugees. Journal of consulting and clinical psychology. 2016;84(7):645

^21^Mölsä M, Punamäki R-L, Saarni SI, Tiilikainen M, Kuittinen S, Honkasalo M-L. Mental and somatic health and pre- and post-migration factors among older Somali refugees in Finland. Transcultural Psychiatry. 2014;51(4):499–525

^22^Knipscheer JW, Kleber RJ. The relative contribution of posttraumatic and acculturative stress to subjective mental health among Bosnian refugees. Journal of Clinical Psychology. 2006;62(3):339–53

^23^Turner SW, Bowie C, Dunn G, Shapo L, Yule W. Mental health of Kosovan Albanian refugees in the UK. The British journal of psychiatry: the journal of mental science. 2003;182:444

^24^Jordans MJ, Semrau M, Thornicroft G, Van Ommeren M. Role of current perceived needs in explaining the association between past trauma exposure and distress in humanitarian settings in Jordan and Nepal. The British Journal of Psychiatry. 2012;201(4):276–81

^25^Norris AE, Aroian KJ. Assessing reliability and validity of the Arabic language version of the Post-traumatic Diagnostic Scale (PDS) symptom items. Psychiatry Research. 2008;160(3):327–34

^26^Kira IA, Templin T, Lewandowski L, Ashby JS, Oladele A, Odenat L. Cumulative Trauma Disorder scale (CTD): Two studies. Psychology. 2012;3(9):643–56

^27^Norris AE, Aroian KJ, Nickerson DM. Premigration persecution, postmigration stressors and resources, and postmigration mental health: a study of severely traumatized U.S. Arab immigrant women.(Report). Journal of the American Psychiatric Nurses Association (JAPNA). 2011;17(4):283–93

^28^Miller KE, Weine SM, Ramic A, Brkic N, Bjedic ZD, Smajkic A, et al. The relative contribution of war experiences and exile-related stressors to levels of psychological distress among Bosnian refugees. Journal of Traumatic Stress. 2002;15(5):377–87

^29^Westermeyer J, Vang T, Neider J. A comparison of refugees using and not using a psychiatric service: An analysis of DSM-III criteria and self-rating scales in cross-cultural context. Journal of Operational Psychiatry. 1983;14(1):36–41

^30^Gammouh OS, Al-Smadi AM, Tawalbeh LI, Khoury LS. Chronic diseases, lack of medications, and depression among Syrian refugees in Jordan, 2013–2014. Prev Chronic Dis. 2015;12:E10

^31^Kira I, Ramaswamy V, Lewandowski L, Mohanesh J, Abdul-Khalek H. Psychometric assessment of the Arabic version of the Internalized Stigma of Mental Illness (ISMI) measure in a refugee population. Transcultural Psychiatry. 2015;52(5):636–58

^32^Lillee A, Thambiran A, Laugharne J. Evaluating the mental health of recently arrived refugee adults in Western Australia. Journal of Public Mental Health. 2015;14(2):56–68

^33^Kessler R, Mroczek D. An update of the development of mental health screening scales for the US national health interview study. Survey Research Center of the Institute for Social Research University of Michigan. 1992

^34^Carta MG, Oumar FW, Moro MF, Moro D, Preti A, Mereu A, et al. Trauma- and stressor related disorders in the tuareg refugees of a cAMP in burkina faso. Clinical practice and epidemiology in mental health: CP & EMH. 2013;9:189

^35^Westermeyer J. Two self-rating scales for depression in Hmong refugees: assessment in clinical and nonclinical samples. Journal of psychiatric research. 1986;20(2):103–13

^36^Hinton DE, Chhean D, Pich V, Pollack MH, Orr SP, Pitman RK. Assessment of posttraumatic stress disorder in Cambodian refugees using the Clinician-Administered PTSD Scale: psychometric properties and symptom severity. Journal of traumatic stress. 2006;19(3):405–9

^37^Veit CT, Ware JE. The Structure of Psychological Distress and Well-Being in General Populations. Journal of Consulting and Clinical Psychology. 1983;51(5):730–42

^38^Hollifield CM, Verbillis-Kolp CS, Farmer CB, Toolson CE, Woldehaimanot CT, Yamazaki CJ, et al. The Refugee Health Screener-15 (RHS-15): development and validation of an instrument for anxiety, depression, and PTSD in refugees. General Hospital Psychiatry. 2013;35(2):202–9

^39^Maneesriwongul W, Dixon JK. Instrument translation process: a methods review. Journal of Advanced Nursing. 2004;48(2):175–86

^40^Hinton WL, Du N, Chen YC, Tran CG, Newman TB, Lu FG. Screening for major depression in Vietnamese refugees: a validation and comparison of two instruments in a health screening population. Journal of general internal medicine. 1994;9(4):202–6

^41^Dinh TQ, Yamada AM, Yee BW. A culturally relevant conceptualization of depression: An empirical examination of the factorial structure of the Vietnamese Depression Scale. International Journal of Social Psychiatry. 2009;55(6):496–505

^42^de Fouchier C, Blanchet A, Hopkins W, Bui E, Ait-Aoudia M, Jehel L. Validation of a French adaptation of the Harvard Trauma Questionnaire among torture survivors from sub-Saharan African countries. European journal of psychotraumatology. 2012;3

^43^Finklestein M, Solomon Z. Cumulative Trauma, PTSD and Dissociation Among Ethiopian Refugees in Israel. Journal of Trauma & Dissociation. 2009;10(1):38–56

^44^Vukcevic M, Momirovic J, Puric D. Adaptation of the Harvard Trauma Questionnaire for working with refugees and asylum seekers in Serbia. Psihologija. 2016;49(3):277–99

^45^Elklit A, Ostergard K, Lasgaard M, Palic S. Social support, coping and posttraumatic stress symptoms in young refugees. Torture. 2012;22(11):11–23

^46^Heeren M, Mueller J, Ehlert U, Schnyder U, Copiery N, Maier T. Mental health of asylum seekers: a cross-sectional study of psychiatric disorders. BMC psychiatry. 2012;12:114

^47^Vinson GA, Chang Z. PTSD symptom structure among West African War trauma survivors living in African refugee camps: A factor analytic investigation. Journal of Traumatic Stress. 2012;25(2):226–31

^48^Ai A, Peterson C, Ubelhor D. War-Related Trauma and Symptoms of Posttraumatic Stress Disorder Among Adult Kosovar Refugees. Journal of Traumatic Stress. 2002;15(2):157–60

^49^Onyut LP, Neuner F, Ertl V, Schauer E, Odenwald M, Elbert T. Trauma, poverty and mental health among Somali and Rwandese refugees living in an African refugee settlement - an epidemiological study. Conflict and health. 2009;3:6

^50^Silove D, Steel Z, McGorry P, Mohan P. Trauma exposure, postmigration stressors, and symptoms of anxiety, depression and post-traumatic stress in Tamil asylum-seekers: Comparison with refugees and immigrants. Acta Psychiatrica Scandinavica. 1998;97(3):175–81

^51^Song S, Kaplan C, Tol W, Subica A, Jong J. Psychological distress in torture survivors: pre- and post- migration risk factors in a US sample. Soc Psychiatry Psychiatr Epidemiol. 2015;50(4):549–60

^52^Robertson CL, Halcon L, Savik K, Johnson D, Spring M, Butcher J, et al. Somali and Oromo refugee women: trauma and associated factors. Journal of Advanced Nursing. 2006;56(6):577–87

^53^Davey C, Heard R, Lennings C. Development of the Arabic versions of the Impact of Events Scale-Revised and the Posttraumatic Growth Inventory to assess trauma and growth in Middle Eastern refugees in Australia. Clinical Psychologist. 2015;19(3):131–9

^54^Hunt N, Gakenyi M. Comparing refugees and nonrefugees: the Bosnian experience. Journal of anxiety disorders. 2005;19(6):717

^55^Matheson K, Jorden S, Anisman H. Relations Between Trauma Experiences and Psychological, Physical and Neuroendocrine Functioning Among Somali Refugees: Mediating Role of Coping with Acculturation Stressors. Journal of Immigrant and Minority Health. 2008;10(4):291–304

^56^Hollifield M, Warner TD, Jenkins J, Sinclair-Lian N, Krakow B, Eckert V, et al. Assessing war trauma in refugees: properties of the Comprehensive Trauma Inventory-104. Journal of traumatic stress. 2006;19(4):527–40

^57^Kira IA, Templin T, Lewandowski L, Ramaswamy V. Collective and personal annihilation anxiety: measuring annihilation anxiety. Psychology. 2012;3(1):90–9

^58^Westermeyer J, Campbell R, Lien R, Spring M, Johnson D, Butcher J, et al. HADStress: a somatic symptom screen for posttraumatic stress among Somali refugees. Psychiatric services. 2010;61(11):1132–7

^59^Gulden A, Westermeyer J, Lien R, Spring M, Johnson D, Butcher J, et al. HADStress screen for posttraumatic stress: replication in ethiopian refugees. The Journal of nervous and mental disease. 2010;198(10):762–7

^60^Powell S, Rosner R, Butollo W, Tedeschi RG, Calhoun LG. Posttraumatic growth after war: A study with former refugees and displaced people in Sarajevo. Journal of Clinical Psychology. 2003;59(1):71–83

^61^Tedeschi RG, Calhoun LG. The posttraumatic growth inventory: Measuring the positive legacy of trauma. Journal of Traumatic Stress. 1996;9(3):455–71

^62^Thulesius H, Hakansson A. Screening for posttraumatic stress disorder symptoms among Bosnian refugees. Journal of Traumatic Stress. 1999;12(1):167–74

^63^Sondergaard HP, Ekblad S, Theorell T. Screening for post-traumatic stress disorder among refugees in Stockholm. Nordic journal of psychiatry. 2003;57(3):185–9

^64^Kim S-H, Goodman GM, Toruno JA, Sherry AR, Kim HK. The Cross-Cultural Validity of the MMPI-2-RF Higher-Order Scales in a Sample of North Korean Female Refugees. Assessment. 2015;22(5):640–9

^65^Dong YLT, Church AT. Cross-cultural equivalence and validity of the Vietnamese MMPI-2: assessing psychological adjustment of Vietnamese refugees. Psychological assessment. 2003;15(3):370–7

^66^Hinton DE, Kredlow M, Pich V, Bui E, Hofmann SG. The relationship of PTSD to key somatic complaints and cultural syndromes among Cambodian refugees attending a psychiatric clinic: The Cambodian Somatic Symptom and Syndrome Inventory (CSSI). Transcultural Psychiatry. 2013;50(3):347–70

^67^Palic S, Carlsson J, Armour C, Elklit A. Assessment of dissociation in Bosnian treatment-seeking refugees in Denmark. Nordic journal of psychiatry. 2015;69(4):307–14

^68^Dao TK, Poritz JM, Moody RP, Szeto K. Development, reliability, and validity of the posttraumatic stress disorder interview for Vietnamese refugees: A diagnostic instrument for Vietnamese refugees. Journal of Traumatic Stress. 2012;25(4):440–5

^69^Clarke G, Sack W, Goff B. Three forms of stress in Cambodian adolescent refugees. An official publication of the International Society for Research in Child and Adolescent Psychopathology. 1993;21(1):65–77

**Table 3:** Brief overview of the self-rated health measurement tools identified in the scoping review

Measurement focus	Measurement tool	Design/use & composition	Publically available	Reliability testing in refugee research	Validity testing in refugee research
General Health	Medical Outcomes Study Short Form (MOS-SF)	Designed for use in clinical practice/research/general pop’n surveys to measure perceived mental/physical health status in past month. Several versions with 6, 12, 20 or 36 items.	Yes	Yes, versions SF-6, SF-12, SF-20 & SF-36	Yes, version SF-12
General Health	New Mexico Refugee Symptom Checklist-121 (NMRSC-121)	Designed for use within non-clinical refugee research for assessment of distressing physical/emotional symptoms in past year (121 items).	No	Yes	Yes
General Health	Personal Wellbeing Index (PWI)	Designed to measure quality of life across cultures in general pop’n (8 items).	Yes	Yes	No
General Health	WHO Quality of Life-BREF (WHOQOL-BREF)	Designed for use in research/clinical settings to assess self-rated quality of life across different cultures in past 2 weeks (26 items).	No	Yes	Yes
General Health	WHO Disability Assessment Schedule 2.0 (WHO-DASS)	Designed for use within clinical/community pop’ns to measure quality of life (36 items).	No	Yes	No
Common Mental Disorders	Afghan Symptom Checklist (ASCL)	Designed to measure psychological distress within non-clinical samples of Afghanis in past 2 weeks (23 items).	No	Yes	No
Common Mental Disorders	Augmented Anxiety Sensitivity Index (AASI)	Designed to measure culturally specific fears within Cambodian refugee outpatients (16 items).	No	Yes	No
Common Mental Disorders	Beck Anxiety Inventory (BAI)	Designed to measure anxiety in adults & adolescents in clinical/research settings (21 items)	Yes	No	No
Common Mental Disorders	Beck Depression Inventory (BDI)	Designed to detect/assess/monitor changes in depressive symptoms in clinical settings (21 items).	Yes	Yes	Yes
Common Mental Disorders	Centre for Epidemiologic Studies-Depression Scale (CES-D)	Designed to measure level of depressive symptoms in a general population in past week (20 items)	Yes	Yes	No
Common Mental Disorders	Depression, Anxiety Stress Scale (DASS)	Designed to measure symptoms of depression/anxiety/stress in community settings in past week (20 items).	Yes	Yes	No
Common Mental Disorders	General Health Questionnaire (GHQ)	Designed for identifying psychiatric illness in Western patients. Several versions with 12, 28, 30 or 60 items.	No	Yes	No
Common Mental Disorders	General Perceived Self-Efficiacy Scale (GPSE)	Designed to assess self-efficacy within Western non-clinical & clinical populations (10 items).	No	Yes	No
Common Mental Disorders	Hopkins Symptom Checklist-25 (HSCL-25)	Originally designed for use in Western clinical settings. Indochinese version developed to screen anxiety & depression in clinical refugee samples (25 items).	No	Yes	Yes
Common Mental Disorders	Hospital Anxiety & Depression Scale (HADS)	Designed for use in Western clinical & research settings to measure anxiety & depression (14 items).	No	No	No
Common Mental Disorders	Internalized Stigma of Mental Illness (ISMI)	Designed to measure internalized stigma within Western clinical samples (29 items)	Yes	Yes	Yes
Common Mental Disorders	Kessler Psychological Scale (KPS)	Developed to screen for distress & mental illness in Western populations in past 4 weeks. Has been adopted in Western & non-Western countries as screening & outcome measure in primary care and mental health settings. Several versions with 6 or 10 items.	Yes	Yes	No
Common Mental Disorders	Mental Health Inventory (MHI)	Designed for use in general Western pop’ns to detect mental health disorders (38 items).	Yes	Yes	No
Common Mental Disorders	Patient Health Questionnaire (PHQ)	Designed to measure depression & severity in Western clinical samples (9 items)	No	No	No
Common Mental Disorders	Refugee Health Screener-15 (RHS-15)	Designed to screen distress, anxiety & depression in clinical samples of refugees (15 items).	Yes	Yes	Yes
Common Mental Disorders	Self-Reporting Questionnaire (SRQ)	Designed as psychiatric screening instrument for use in primary health care/community settings in low/middle income countries. Several versions with 5 or 20 items.	Yes	No	No
Common Mental Disorders	Sheenan Disability Inventory (SDI)	Designed to assess functional impairment associated with anxiety disorder diagnosis in Western clinical samples (3 items)	Yes	No	No
Common Mental Disorders	Symptom Checklist-90 (SCL-90)	Designed as mental health screening tool for Western clinical samples (90 items; 10 subscales asking about mental health symptoms in past week).	No	No	Yes
Common Mental Disorders	Vietnamese Depression Scale (VDS)	Designed to measure depression among clinical samples of refugees (15 items)	No	Yes	Yes
Trauma/PTSD	Annihilation Anxiety Scale (AAS)	Designed for use in clinical settings & in research with severely traumatized populations (eg refugees). (3 items)	No	Yes	Yes
Trauma/PTSD	Cambodian Somatic Symptom & Syndrome Inventory (CSSI)	Designed for use in clinical samples of Cambodian refugees to assess somatic symptoms & cultural syndromes in past week (23 items). Very specific to Cambodians.	No	Yes	Yes
Trauma/PTSD	Comprehensive Trauma Inventory-104 (CTI-104)	Designed for use within community samples of refugees to assess traumatic events (104 items).	Yes	Yes	Yes
Trauma/PTSD	Cumulative Trauma Disorder Scale (CTDS)	Designed for use within clinical & non-clinical samples of refugees to measure trauma symptoms in past month (15 items)	No	Yes	Yes
Trauma/PTSD	Dissociative Experiences Scale (DES)	Designed to measure dissociation in general & clinic Western populations (28 items).	Yes	Yes	No
Trauma/PTSD	HADStress	Designed to measure somatic symptoms within community sample of refugees (4 items).	No	No	Yes
Trauma/PTSD	Harvard Trauma Questionnaire (HTQ)	Designed for clinical samples of refugees to measure trauma. Must be modified and adapted to the characteristics of each cultural group. 4 parts. Part 1 measures trauma. Part 2 asks for description of most traumatic experience/event experienced during the refugee experience. Part 3 assesses likelihood of head injury. Part 4 measures trauma symptoms.	No	Yes	Yes
Trauma/PTSD	Health Leaflet	Designed to measure PTSD within community samples of refugees (15 items).	No	No	Yes
Trauma/PTSD	Impact of Events Scale-Revised (IES-R)	Designed to measure current subjective distress related to a specific event among Western clinic samples (15 items).	Yes	Yes	Yes
Trauma/PTSD	Minnesota Multiphasic Personality Inventory (MMPI)	Designed to assess personality traits & psychopathology among Western clinic samples (567 items).	Yes	No	Yes
Trauma/PTSD	Post Migration Living Difficulties Scale (PMLD)	Designed to assess current life stressors of asylum-seekers (23 items).	No	No	Yes
Trauma/PTSD	Post Traumatic Growth Inventory (PTGI)	Designed to assess positive outcomes among traumatized non clinic Western pop’ns (21 items).	Yes	Yes	Yes
Trauma/PTSD	Posttraumatic Diagnostic Scale (PDS)	Designed to measure self-report measure of PTSD in clinical/research settings. 4 parts. Part 1 is checklist of traumatic events experienced/witnessed by respondent. Part 2: asks respondents to indicate which event has disturbed them the most in past month. Part 3 asks for PTSD symptoms items experienced in past month.	Yes	Yes	Yes
Trauma/PTSD	Posttraumatic Stress Disorder Interview for Vietnamese Refugees (PTSD-IVR)	Designed to assess trauma among clinic samples of Vietnamese refugees (30 items)	No	Yes	Yes
Trauma/PTSD	Posttraumatic Symptom Disorder Checklist-90 (PCL-90)	Designed to measure PTSD among non-clinical samples in past month (17 items)	Yes	No	Yes
Trauma/PTSD	Posttraumatic Symptom Scale (PTSS)	Designed to measure PTSD symptoms in past week within trauma affected samples (10 or 16 items)	Yes	Yes	No
Trauma/PTSD	Primary Care Post-Traumatic Stress Disorder Screen (PC-PTSD)	Designed for use in primary care/other medical settings to assess dimensions of PTSD (5 items).	Yes	No	No
Trauma/PTSD	Resettlement Stressor Scale (RSS)	Designed within a community sample of Cambodian refugee adolescents to assess trauma events in migration/present settlement (32 items).	Yes	No	Yes
Trauma/PTSD	Traumatic Life Events Questionnaire (TLEQ)	Designed in general clinic western population for use in research/primary care/emergency rooms to assess potentially traumatic events (23 items).	No	No	No
Trauma/PTSD	War Trauma Scale (WTS)	Designed within clinical samples of Cambodian adolescent refugees that had lived through the Pol Pot regime. Items are all worded in relation to Pol Pot era. (42 items).	No	Yes	Yes

### Characteristics of measurement tools

The 45 measurement tools were used to measure general health, common mental disorders and trauma/PTSD among refugees. In terms of measurement focus, general health was measured using five tools; 19 tools assessed common mental disorders, such as anxiety and depression; and 21 tools investigated trauma/PTSD (see Tables 2 and 3). No consensus on the preferential use of different measurement tools emerged; although the most widely used tools across the studies were the Harvard Trauma Questionnaire for the assessment of trauma/PTSD and the Hopkins Symptom Checklist-25 for anxiety and depression. Sixteen of the identified measurement tools were developed specifically within samples of refugees and were used in half of the reviewed studies.

Aside from the Afghan Symptom Checklist, which was developed within a community sample in Afghanistan; the remaining 28 tools were designed for use in Western populations 12 of these Western developed tools were designed to be used in a clinical environment, but were frequently used in community studies of refugee populations. For example, over half of the community based studies used at least one tool that had been specifically developed for use within Western clinical populations. Translation of the Western designed tools mostly involved back-translation (85 studies). Twenty two studies reported translating English versions of measurement tools word-for-word into the refugee language. Pre-translated versions of the measurement tools were used in 28 of the articles reviewed and 15 studies reported using bilingual interpreters were used to verbally translate the tool items to the target population.

The pre-determined cut-off scores of measurement tools were routinely applied across most studies in spite of the conditions of their original development. Only two studies were identified that adjusted the cut-off score of their selected measurement tools to suit their target population [35, 36]. Measurement tools for use in non-clinical environments must be brief, easy to use and interpret, cost-effective and accessible. For example, the Minnesota Multiphasic Personality Inventory is designed for use in clinical samples, yet was used in several community studies of refugees [37, 38]. This tool is 567 items long, takes 60–90 min to complete and requires specialist training to administer and score.

### Statistical testing of measurement tools

More than half of the 45 tools have been evaluated statistically for reliability and validity among refugee populations. Reliability data was reported for 33 of the tools and 24 tools had published validation data (see Tables 2 and 3). Twenty tools have published reliability *and* validity data among refugee populations. Prior to the year 2000 only seven of the tools described in this review had evidence of reliability testing and validity within refugee populations. A notable finding is that eight of the tools have published reliability data in refugee groups, but no published reliability data. A measurement tool cannot be valid unless it is reliable [39] and reasons for the absence of reliability data for these tools could not be ascertained in this review. Six of the reviewed tools have not been tested for either validity or reliability among refugees, but meet the criteria of being used in refugee research where the sample size was 150 participants or greater. Eighteen tools were validated by one study each and six tools were validated by two or more studies (see Table 2).

The methods used to conduct reliability and validity testing within studies was highly variable and there appeared an emphasis on conducting reliability testing rather than validation testing of measurement tools. In terms of reliability testing, internal consistency the most commonly reported statistic while test-retest reliability and inter-rater reliability was mostly overlooked. Test-retest reliability was only reported in studies using 11 tools and inter-rater reliability was reported in only 2 studies. Where the validation testing of measurements was conducted, there were inconsistencies across studies in the extent of testing. Ideally, refugee health measures should have, at a minimum, confirmed content and construct validity within refugee populations; however this was only observed in a few of the studies. Where a tool was adapted for refugee research, there appeared an emphasis on reliability and criterion validation. Testing only criterion validity does not assess whether the measure appropriately captures culture specific constructs, such as depression, anxiety or PTSD.

Where the development of a measurement tool was described, few studies described efforts to conduct a qualitative analysis of the concepts being measured in the development of the tools; incorporating ten tools. These tools were all designed specifically for use in refugee research, with the exception of the Hopkins Symptom Checklist-25 (Indochinese version) and Medical Outcomes Study Short Form −12, which have been adapted for use in refugee populations.

### Modification of measurement tools

Eight of the 44 measurement tools underwent some form of modification to make them more culturally appropriate. These modifications included addition/removal of items [40–46] and cut-off score modification [35, 36]. The Harvard Trauma Questionnaire was the most commonly modified tool, and is in accordance with the authors’ recommendation that the tool be modified and adapted to the characteristics of each cultural group [40]. Fourteen of the 62 studies which used the Harvard Trauma Questionnaire modified the tool to be more appropriate for their target refugee population.

The translation of measurement tools across studies was variable. Ideally, translation of measures should undergo a standard translation/backtranslation process to ensure semantic and conceptual equivalence [47] and to avoid culturally sensitive material. In less than half of the studies reviewed, there was evidence that the researchers had undertaken thorough back-translation of the measurement tool(s). A number of studies reported translating English versions of measurement tools word-for-word into the refugee language, which is problematic in cross cultural research as the questions or items may not be communicated correctly, especially if any idioms were used in the source language [48]. Attempts to establish conceptual and/or thorough linguistic equivalence of only 11 tools was identified.

## Discussion

This scoping review identified that 45 different self-rated health measurement tools were used to measure self-rated health in refugee populations. Most of the 183 studies detected in the review study were cross sectional explorations of the mental health status of refugees living in community settings in Western nations. A third of the tools were designed specifically for use within refugee populations. More than half of the measurement tools have been evaluated for reliability and/or validity within refugee populations.

Apparent from this review is that no consensus exists on the use of different measurement tools amongst researchers and there are no standard criteria against which quality assessment of these tools can be made. This has resulted in a large number of tools of varying rigour being used in refugee health research. The resulting variability in structure, reliability and validity increases the potential for inaccurate conclusions to be made concerning the health of refugee populations. Several studies have previously reported large variations in prevalence rates of mental health disorders among refugees [4, 8, 14, 49] and these disparities have been attributed to inconsistencies in methods and measurement tools used for data collection, analysis, and reporting [50]. Tools used in refugee health research should have at a minimum, demonstrated reliability and validity of the construct within refugee populations to ensure that cultural concepts and constructs are able to be accurately measured [26, 51]. A number of tools identified in this review have published reliability and validity within refugee groups, but few provided evidence of testing for construct validity and the methods used to confirm reliability and validity were highly variable across studies.

Refugee self-rated health measurement tools are also frequently used out of the context for which they were designed. For example, clinical tools are developed for use among treatment seeking patients and are designed to be administered in safe and trusting environments where care can be provided for any adverse reactions. They are not specifically designed for non-clinical settings where the level of contact between respondents and often non-clinical, administrators is brief. Clinically developed tools also have established cut off points for identifying individuals who are identified as being positive for specific disorders and these may shift as the setting changes from a clinic to a community sample. It is not known whether the application of measurement tool scores across heterogeneous populations leads to reasonable inferences concerning symptom severity and diagnoses [47, 51], but there is a body of work suggesting that using a single cut-off score may not be a valid procedure for cross cultural samples [51–55].

The challenges in developing and adapting health measurement tools for refugee research were evident in the reviewed literature. The methods used were inconsistent and/or limited across studies. For example, testing only reliability was common across the reviewed studies. Reliability only addresses the degree to which measurement tools result in reproducible results across different interviewers and applications [56]; it does not measure the degree to which the tool actually measures the construct of interest. Where validity testing was conducted, there was a focus on criterion validity (or ‘caseness’). Criterion validity does not assess whether the measure appropriately captures culture specific constructs, such as depression, anxiety or PTSD. Determining the construct validity of a tool demonstrates how well the tool measures the constructs it was designed to measure [34] and ensures that inferences made using the results of such assessments, such as severity of symptoms and prevalence rates are supported. For example, the PTSD symptom patterns of refugees may deviate from those of Western populations because of both cultural and war-related factors, as well as post-traumatic life circumstances [53, 57]. The failure to appropriately standardize or adapt existing measures for use with refugee populations means that they may lead to incorrect generalizations about the health of refugees and this can have a widespread effect and can lead to the development and implementation of incorrect interventions and policies [58].

This review found inconsistencies in the translation of measurement tools across studies. Translation should be undertaken using thorough back-translation of the measurement tool(s) into the first language of those being assessed by using the back-translation method by itself or in combination with a committee or bilingual assessment method [47, 59]. The translation of English versions of measurement tools word-for-word into the refugee language is problematic in cross cultural research as the questions or items may not be communicated correctly, especially if any idioms were used in the source language [48, 50]. Appropriate translation ensures ensuring semantic and conceptual equivalence and is one of the requirements for establishing validity [47, 60].

Health measurement tool items should also be examined to illustrate why they are culturally relevant in terms of the rationale behind their inclusion and understanding what it means for a person in that culture to have the symptom or syndrome or how they vary across cultures. For example, the way in which western psychology describes PTSD does not fit the symptoms of people from non-western cultures, yet tools measuring trauma in refugees often use western trauma concepts and constructs. The exploration and establishment of equivalence of measurement tools between local indigenous constructs and symptoms is an important step in ensuring that the measurement tools are tapping into the respondents’ understanding of their health [26, 61] and was frequently overlooked in the reviewed refugee research studies.

## Recommendations

Researchers would benefit from the development of guidelines to instruct proper and consistent measurement design and testing; such as the achievement of cultural equivalency across health concepts, reliability and validity across refugee population groups and settings. For example, researchers would benefit from the use of standardized procedures such as the Translation Monitoring Form [62] which provides a method for the systematic translation and adaptation of measurement tools. Evaluation of the performance of measurement tools needs to be undertaken in rural and remote settings. Given that receiving nations, such as Australia and the United States of America (USA), are resettling refugees beyond the major metropolitan regions, it is important that these populations are not overlooked so that we can understand the ongoing health needs of remote refugee populations to inform often limited, and often over-stretched, rural and regional health services.

The development of integrated and comprehensive measurement tools to assess all elements of relevance to the health of refugees would be beneficial to health service providers. The current tools are not comprehensive, but rather assess parts of experiences and/or symptoms and disorders. Measures, such the *Refugee Mental Health Assessment Package*, hold promise as an integrated and comprehensive package of measures to assess all elements of relevance to the mental health of refugees [63].

Currently, there is an underrepresentation of tools measuring resettlement stressors, pre-war, pre-conflict and non-conflict trauma within refugees. The resettlement environment is significant to the health of refugees and the lack of measurement tools to capture this information means that the development of health interventions during resettlement is challenging. Many refugees may experience trauma prior to the war or conflict-related event that can causes them to flee their home. This trauma could be due to religious or political oppression which then resulted in the outbreak of civil or international war. Therefore, these refugees are traumatised prior to displacement, yet there are no assessments to measure this pre-war or pre-conflict trauma. This represents a significant gap in refugee research and research is required into the development of such measures.

Optimal tools for the measurement of refugee self-rated health are those that have reported reliability and validity testing in refugee populations. Several tools fit these criteria and include the Medical Outcomes Study Short Form and New Mexico Refugee Symptom Checklist-121 for general health assessment; the Hopkins Symptom Checklist-25 and Refugee Health Screeners-15 for common mental disorders; and the Harvard Trauma Questionnaire and Comprehensive Trauma Inventory-104 for the assessment of trauma/PTSD within refugees (see Tables 2 and 3). Also, tools should not be used out of context for which they were designed. Measurement tools designed for use in clinical settings may not be suitable for use in community environments and vice-versa (see Tables 2 and 3).

## Limitations

There are several limitations to this review. Firstly, electronic searches of the literature are not error free, and citations to some studies and measurement tools may not be included in the literature. In addition, our review will be limited by the fact that the searches were limited to articles in English published since 2000. However, given the limited volume of research in this area, the search for validation studies of tools utilised in refugee research was not limited by date of publication. The results of the review should be interpreted with the knowledge that scoping reviews do not screen for quality of studies and, therefore, they include studies with large variations in study methodologies.

## What does this study add to the literature?

The results of this review have important research, policy and practice implications, which are outlined below.

### Research implications

This review study provides an up to date compilation of contemporary general health, common mental disorders and trauma/PTSD assessments that rely on client self-report. We have identified a number of measurement tools that had been evaluated for reliability and/or validity since the publishing of prior reviews and this included a number of newly developed tools as well as those used to assess general health.

### Policy implications

Given the increasing importance of patient measured outcomes, our results provide (particularly in Table 2) a compendium of self-report measurements for policy makers to use for program evaluation.

### Practice implications

A number of jurisdictions are seeking input from patients on the quality and accessibility of health care services. A number of the measurement tools would be highly relevant for clinicians to identify performance as part of quality improvement.

## Conclusion

Tools for use in refugee health research should have demonstrated reliability and validity in refugee populations to ensure accurate measurement of the health concept(s) under investigation. Consideration should also be given to the setting in which the tool was originally designed. This review shows that there are currently a number of reliable and valid measurement tools available for use in refugee health research which can be used across a variety of settings. However, further work is required to achieve consistency in tool quality and in the use of these tools. Methodological guidelines are required to assist researchers and clinicians in the development and testing of subjective health measurement tools. In the interim, an achievable and very useful study would be a comprehensive evaluation of the most current and robust self-rated health measurement tools for use within refugee health research.

## Abbreviations

PRISMA: Preferred reported items in systematic reviews and meta-analysis
PTSD: Post-traumatic stress disorder
UNHCR: United Nations High Commission for Refugees
USA: United States of America
